# *Toxoplasma gondii* seroprevalence in extensively farmed wild boars (*Sus scrofa*) in Denmark

**DOI:** 10.1186/s13028-019-0440-x

**Published:** 2019-01-15

**Authors:** Celine Kaae Laforet, Gunita Deksne, Heidi Huus Petersen, Pikka Jokelainen, Maria Vang Johansen, Brian Lassen

**Affiliations:** 1 Department of Veterinary and Animal Sciences, University of Copenhagen, Grønnegårdsvej 15, 1870 Frederiksberg C, Denmark; 2 Institute of Food Safety, Animal Health and Environment, “BIOR”, Lejupes Str. 3, Riga, Latvia; 3 Faculty of Biology, University of Latvia, Jelgavas Str. 1, Riga, Latvia; 4 Section for Diagnostics and Scientific Advice, National Veterinary Institute, Technical University of Denmark, Kemitorvet, 2800 Kgs. Lyngby, Denmark; 5 Laboratory of Parasitology, Department of Bacteria, Parasites & Fungi, Infectious Disease Preparedness, Statens Serum Institut, Artillerivej 5, 2300 Copenhagen S, Denmark; 6 Faculty of Veterinary Medicine, University of Helsinki, P.O. Box 66, 00014 Helsinki, Finland; 7 Institute of Veterinary Medicine and Animal Science, Estonian University of Life Sciences, Kreutzwaldi 62, 51014 Tartu, Estonia

**Keywords:** Serology, *Sus scrofa*, *Toxoplasma gondii*, Wild boar, Zoonosis

## Abstract

*Toxoplasma gondii* is a zoonotic parasite of worldwide importance. In this study, we estimated *T. gondii* seroprevalence in extensively farmed wild boars in Denmark, where little is known about *T. gondii* in animal hosts. Our study focused on wild boars because they are considered good indicator species for the presence of *T. gondii*, and wild boar meat is used for human consumption. Serum samples from 101 wild boars collected in 2016–2018 from five different locations from the continental part of Denmark, Jutland, were screened for anti-*T. gondii* antibodies. The samples were analysed using a commercial indirect enzyme-linked immunosorbent assay (ELISA). Samples from 28 (27.7%) of the 101 wild boars tested positive with the ELISA. The odds for a wild boar to test seropositive were higher if it was sampled during the hunting season 2017–2018 than during 2016–2017 and if it was reported to be at least 1 year old than if it was younger (logistic regression model with the two variables: odds ratios 17.5 and 3.9, respectively). A substantial proportion of the investigated extensively farmed wild boars had been exposed to *T. gondii*. Moreover, the parasite appeared widespread, at least in the continental part of Denmark, Jutland, as seropositive wild boars were found from all five sampled locations. Assuming seropositivity indicates hosting viable parasites, consumption of undercooked wild boar meat from Denmark is a potential source of *T. gondii* infections to other hosts, including humans.

## Findings

The zoonotic protozoan parasite *Toxoplasma gondii* has a wide host range [1], including wild boars (*Sus scrofa*). Wild boars are considered good indicator hosts for the presence of *T. gondii*, and seroepidemiological studies have shown that farmed and free-ranging wild boars are commonly exposed to *T. gondii* in several countries [2, 3]. *Toxoplasma gondii* infections of wild boars are typically subclinical, but clinical congenital toxoplasmosis has been reported [4]. More importantly, *T. gondii* infections of wild boars are of public health importance, because wild boars are farmed and hunted for human consumption. Thus, undercooked tissues of wild boars that are infected with *T. gondii* may serve as infection source to humans. Among zoonotic foodborne parasites of greatest concern, *T. gondii* has been ranked 4th globally [5] and 2nd in Europe [6].

In Denmark, free-ranging wild boars are currently not welcomed. However, wild boars are extensively farmed in fenced areas where hunters regulate the population during hunting seasons. In the Danish Central Livestock Register, altogether 63 fenced areas are listed for keeping wild boars [7]. *Toxoplasma gondii* has recently gained attention on the human medical side in Denmark [8, 9]. However, major knowledge gaps in the local epidemiology of this zoonotic parasite exist, as only few studies have focused on animal hosts [10]. Literature searches identified no previous studies on *T. gondii* in wild boars from Denmark.

The aim of this seroepidemiological study was to estimate *T. gondii* seroprevalence in extensively farmed wild boars in Denmark. The sampling was a convenience sample from five separate locations in the continental part of Denmark, Jutland. The wild boars lived in fenced outdoor areas where they received supplementary feeding. The sampling took place during two hunting seasons, from October 2016 to January 2017 and from October 2017 to January 2018. The blood samples were collected post mortem from legally hunted wild boars. Sera were separated by centrifugation and stored at − 21 °C until the analysis.

The samples were analysed for antibodies against *T. gondii* using a commercial indirect enzyme-linked immunosorbent assay (ELISA; ELISA ID Screen Toxoplasmosis Indirect Multi-species, IDvet, Grabels, France), following the instructions of the manufacturer. The samples and the controls provided in the kit were analysed in duplicate. The optical density (OD) was read at 450 nm. Results were evaluated by calculating the S/P% (sample/positive percentage) = (mean OD of sample − mean OD of negative control)/(mean OD of positive control − mean OD of negative control) × 100. Samples with S/P% ≤ 40% were considered negative, 40–50% doubtful, and ≥ 50% positive.

The outcome was dichotomised: wild boars that tested positive with the ELISA were considered seropositive; others were considered seronegative. The animal-level variables evaluated were ‘season’ (sample collected in 2016–2017 vs. 2017–2018), ‘age group’ (reported to be < 1 year old vs. at least 1 year old) and ‘sex’ (female vs. male). The locations were coded 1–5, and evaluated as dummy variables ‘location’. Each variable was initially analysed alone (crude, univariable analysis). Secondly, all four variables were included in a logistic regression model, followed by step-wise removal until only significant variables and confounders were left.

OpenEpi [11] was used to evaluate sample size, to calculate confidence intervals (CI) for the proportions (Mid-P Exact), and to compare proportions (e.g. the seroprevalence in the two age groups; our seroprevalence estimate vs. estimates reported in other studies) using two-by-two tables (2-tailed *P* value, Mid-P Exact). Logistic regression analyses were performed using Stata 13.1 (StataCorp, College Station, TX, USA). P-values (Mid-P exact; those calculated by Stata) < 0.05 were considered significant.

The minimum needed sample size for estimating the seroprevalence was calculated to be 85–91 animals, based on the seroprevalence estimates of 33% and 50% from Finland and Sweden, respectively [12, 13], a 80% confidence level, and a population size of 200 (estimated annual wild boar hunting bag in Denmark). The sample size available for the study was 101 wild boars, 38 (37.6%) of which were reported to be younger than 1 year old. Altogether 53 (52.5%) were female and 47 (46.5%) were male (Table 1). Age group was not reported for two wild boars, and sex was not reported for one wild boar.

**Table 1:** *Toxoplasma gondii* seroprevalence in extensively farmed wild boars (*Sus scrofa*) in Denmark, by hunting season, age group, sex, and location

	No. of animals	No. of seropositive animals	Seroprevalence (%)	95% confidence interval
Season				
2016–2017	41	2	4.9	0.8–15.2
2017–2018	60	26	43.3	31.3–56.0
Age group (years)				
< 1	38	6	15.8	6.7–30.0
≥ 1	61	22	36.1	24.8–48.6
Sex				
Female	53	19	35.8	23.8–49.4
Male	47	9	19.1	9.8–32.2
Location				
Location 1	32	7	21.9	10.1–38.6
Location 2	14	6	42.9	19.6–68.9
Location 3	10	4	40.0	14.2–70.9
Location 4	26	7	26.9	12.6–46.1
Location 5	19	4	21.1	7.1–43.3
Total	101	28	27.7	19.7–37.1

Age group was unknown for two wild boars, sex was unknown for one wild boar

Samples from 28 (27.7%, 95% CI 19.7–37.1) of the 101 wild boars tested positive with the ELISA. The manufacturer’s cut-off values for considering the test as validated were met. Four wild boars tested doubtful (considered seronegative in the interpretation of results).

The seroprevalence estimate was lower in 2016–2017 than in 2017–2018 (Table 1, P-value < 0.001). Based on univariable model, the odds to test seropositive were 14.9 (95% CI 3.3–67.5) times higher in wild boars sampled in 2017–2018 than in those sampled in 2016–2017. The seroprevalence estimate in wild boars reported to be at least 1 year old was significantly higher than the estimate in reportedly younger wild boars (Table 1, P-value < 0.05). Based on univariable model, the odds to test seropositive were 3.0 (95% CI 1.1–8.3) times higher in wild boars reported to be at least 1 year old than in reportedly younger wild boars. The seroprevalence estimate was 35.8% in females and 19.1% in males; this difference was not statistically significant. Several seropositive wild boars were found from all five locations (Table 1). The variables ‘sex’ and ‘location’ were omitted from the multivariable model as non-significant; the final model used 99 observations (those with data available for the variables) and contained the variables ‘season’ and ‘age group’ (odds ratio 17.5, 95% CI 3.7–81.6, and odds ratio 3.9, 95% CI 1.3–11.8, respectively).

This study is the first to report seroprevalence of *T. gondii* in wild boars in Denmark and documents a wide exposure among extensively farmed wild boars. The results add to the scarce knowledge on *T. gondii* in its animal hosts in Denmark.

The seroprevalence estimate of 27.7% was obtained using an ELISA kit intended for samples from several host species, including pigs. However, the sensitivity and specificity are not reported. Partly because the seroprevalence estimate for 2016–2017 was unexpectedly low (Table 1), we tested the 41 samples from that hunting season also using a commercial modified direct agglutination test (DAT; Toxo-Screen DA, bioMérieux, Marcy-l’Étoile, France; samples diluted 1:40). Samples from four (9.8%, 95% CI 3.2–21.9) of the 41 wild boars tested positive with the DAT. The DAT-results from each sample and its duplicate were consistent. The four DAT-positive samples comprised both ELISA-positive samples, one sample that tested negative with the ELISA, and one sample that tested doubtful with the ELISA. The DAT has no host-species-specific reagents and it has been widely used for both domestic pigs and wild boars. DAT detects only specific immunoglobulin G antibodies, because possible immunoglobulin M antibodies are denatured by 2-mercaptoethanol, and very recent infections where these antibodies are not yet formed are missed [2]. The DAT-titer of 40, which we used as cut-off to define seropositivity can be considered high [1, 14], and thus even the DAT-based estimate (which for these 41 samples is the same as if the two methods would have been used in parallel, which increases sensitivity) may underestimate the infection prevalence. Infective *T. gondii* has been isolated from wild boars with lower DAT-titers than our cut-off for seropositivity [14, 15]. Further, we ruled out the possibility that the difference could have been due to clustering by location. Three of the five locations were sampled during both hunting seasons, and the results from this subgroup were similar to the overall results (n = 77, ELISA-seroprevalence 2/41, 4.9%, 95% CI 0.8–15.2 in 2016–2017 and 16/36, 44.4%, 95% CI 29.0–60.8 in 2017–2018, P-value < 0.001, univariable OR 15.6, 95% CI 3.3–74.7). Monitoring the situation over several years could be useful to investigate the annual variation and, in particular, whether our study with two sampling time points identified a true increase in seroprevalence.

The seroprevalence was significantly higher in the wild boars reported to be at least 1 year old than in those reported to be younger. This result is in line with the result from free-ranging wild boars from Sweden [13], and indicates that the infections are acquired: older wild boars have had longer time to encounter the parasite. Whether the antibodies persist lifelong in wild boars has been questioned [16]. Hence, negative results, in particular in older wild boars, may not rule out earlier exposure, and the estimate in the older age group may be an underestimation of the proportion exposed. Regardless, it is also noteworthy that the seroprevalence estimate in wild boars reported to be below 1 year of age was already relatively high, 15.8%, as this indicates substantial infection pressure. In the Nordic-Baltic region, Estonia is a country where *T. gondii* infection pressure appears particularly high, and there, no statistically significant difference in seroprevalence between age groups was observed in free-ranging wild boars (22.4% in younger age group and 27.6% in older age group) [17].

In this study, *T. gondii* seroprevalence was 1.9 times higher in females than in males, but the difference was not statistically significant. In farmed wild boars in Finland, the difference was similar, 1.9-fold and significant, and females had significantly higher odds to test seropositive than males also based on the results of a multivariable random effects logistic regression model [12]. Whether and why female wild boars could be more exposed to the parasite on the farms than male wild boars would merit further studies.

Seropositive wild boars were detected from all five sampled locations in the continental part of Denmark, Jutland, but the results may not represent the situation in other parts of Denmark nor the environment outside the fenced areas.

A recent systematic review and meta-analysis on *T. gondii* in wild boars estimated the seroprevalence to be 23% globally and 26% in Europe [3]. Our overall estimate (27.7%) was thus of the expected magnitude, and did not differ significantly from those reported for farmed wild boars in Finland (33.0%) [12] and Latvia (20.3%) [18] nor from those reported for free-ranging wild boars in Latvia (35.1%) [18], Estonia (24.0%) [17], and Sweden (28.6%) [19], but it was lower (P-value < 0.001) than an earlier estimate for free-ranging wild boars in Sweden (49.5%) [13]. It should be emphasized that the results of different studies are not directly comparable due to different farm management and game management, different sampling strategies, and different methods applied, but they illustrate that the parasite is common and endemic in the Nordic-Baltic region.

Because wild boars are considered good indicators for *T. gondii*, our results suggest the zoonotic parasite is common and widespread in the extensive wild boar farming environment in the continental part of Denmark, Jutland. Because *T. gondii* seropositivity indicates exposure, chronic infection, and carrying infective parasites in the tissues, eating undercooked meat from wild boars from Denmark may present *T. gondii* infection risk to other hosts, including humans.

## Abbreviations

CI: confidence interval
DAT: modified direct agglutination test
ELISA: enzyme-linked immunosorbent assay
OD: optical density
S/P: sample/positive
